# Application effects of remimazolam and propofol on elderly patients undergoing hip replacement

**DOI:** 10.1186/s12871-022-01641-5

**Published:** 2022-04-23

**Authors:** Junbao Zhang, Xin Wang, Qing Zhang, Zicheng Wang, Shoufeng Zhu

**Affiliations:** Department of Anesthesiology, The Second People’s Hospital of Hefei, Hefei Hospital Affiliated to Anhui Medical University, Guangde Road, Yaohai District, Hefei City, Anhui Province China

**Keywords:** Remimazolam, Propofol, Hip replacement, Elderly

## Abstract

**Objective:**

To explore the anesthetic and analgesic effects of remimazolam and propofol in elderly patients undergoing hip replacement and their effects on respiratory and circulatory systems, stress and cognitive function.

**Methods:**

Sixty elderly patients undergoing elective hip replacement in the hospital were selected as the research subjects, and they were divided into the remimazolam group and the propofol group according to the admission sequence of patients. The remimazolam group was anesthetized with remimazolam, and the propofol group was anesthetized with propofol. The anesthesia-related indicators, perioperative pain degree [Visual Analogue Scale (VAS)], circulatory indicators [heart rate, mean arterial pressure (MAP)] before anesthesia (T_0_), immediately before laryngeal mask insertion (T_1_), at 5 min after laryngeal mask insertion (T_2_), at 30 min after laryngeal mask insertion (T_3_) and at 5 min after laryngeal mask removal (T_4_), stress response indicators (plasma epinephrine, norepinephrine, cortisol) before anesthesia induction and at 24 h and 72 h after surgery, cognitive function [Mini-Mental State Examination (MMSE)] and adverse reactions were compared between the two groups.

**Results:**

Among the 60 enrolled patients, only 1 case was excluded due to withdrawal, thus 30 cases in the remimazolam group and 29 cases in the propofol group were included. There were statistically significant differences in the heart rate, MAP, plasma epinephrine, norepinephrine, cortisol and VAS score in the two groups from the aspects of interaction effect and time-point effect (*P* < 0.05). The heart rate and MAP at T_1_, T_2_ and T_3_ in the two groups were significantly decreased compared with those at T_0_, but the heart rate and MAP in the remimazolam group at T_1_, T_2_ and T_3_ were significantly higher than those in the propofol group (*P* < 0.05). There were no statistical differences in the anesthesia time, awakening time and extubation time between the remimazolam group and the propofol group (*P* > 0.05). The levels of plasma epinephrine, norepinephrine and cortisol in the two groups were significantly higher at 24 h and 72 h after surgery than those before anesthesia induction, and the above levels were significantly lower in the remimazolam group than those in the propofol group (*P* < 0.05). The VAS scores at each time point in the two groups were significantly reduced compared to before surgery, but there was no statistically significant difference between the two groups after surgery (*P* > 0.05). The MMSE scores of the two groups were significantly lower at 1 d and 3 d after surgery compared with those before anesthesia induction, but the score in the remimazolam group was significantly higher than that in the propofol group (*P* < 0.05). In addition, the incidence rates of adverse reactions were significantly lower in the remimazolam group compared to the propofol group (*P* < 0.05).

**Conclusion:**

Compared with propofol, remimazolam can achieve equivalent anesthetic and analgesic effects in elderly patients undergoing hip replacement. However, the latter one can significantly relieve respiratory and circulatory suppression, stress response and cognitive dysfunction, with good safety.

**Trial registration:**

This single-center, prospective, RCT has completed the registration of the Chinese Clinical Trial Center at 31/12/2021 with the registration number ChiCTR2100055039.

Hip replacement can effectively relieve the joint pain, correct the deformity and recover and improve the joint motor function of patients, thus it is the most effective surgical intervention for elderly patients with hip diseases [1, 2]. According to statistics, the incidence rate of postoperative cognitive dysfunction in elderly surgical patients is as high as 38% [3], posing a serious threat to the surgical effects and prognosis of patients. Elderly patients with hip replacement, as a special group, have low body resistance and are often complicated with multiple underlying diseases, so they have high requirements for perioperative anesthesia and most of them prefer general anesthesia [4, 5]. Propofol has been widely used in general anesthesia due to its rapid onset of action, short action time and fast metabolism, but on the other hand it also has limitations, such as a narrow therapeutic index and significant inhibitory effects on circulation and respiration, which may increase the surgical risk for elderly patients [6]. Remimazolam is a new type of benzodiazepine anesthetic drug. Doi, M., et al. demonstrated that remimazolam was well tolerated and non-inferior to propofol with regard to efficacy as a sedative hypnotic for general anesthesia which is suitable for clinical application of general anesthesia [7]. Chen et al. [8] indicated that benzenesulfonic remimazolam and propofol could have equivalent anesthetic effects in colonoscopy diagnosis and treatment, but the former one was significantly safer than the latter one. It can be seen that remimazolam and propofol have been clinically studied in the induction and maintenance of general anesthesia, but there is still no clear conclusion about the application effects of the two drugs in elderly patients undergoing hip replacement. This study adopts remimazolam and propofol to maintain anesthesia of 60 elderly patients undergoing elective hip replacement, and compares the differences in circulatory indicators, stress indicators, anesthetic effects and perioperative pain degree of the two so as to provide more reference for choosing a safe and effective anesthesia regimen for elderly patients undergoing hip replacement.

## Data and methods

### Research subjects

Sixty elderly patients who underwent elective hip replacement in the hospital between April 2021 and July 2021 were enrolled as the research subjects. (1) Inclusion criteria included presence of hip destruction confirmed by X-ray or CT imaging; conforming to indications for hip replacement determined by Chinese Medical Association Orthopaedic Society (① ineffective conservative treatment of hip osteoarthritis, ② obviously limited joint function affecting life, ③ femoral head collapse in femoral head necrosis due to a variety of reasons, ④ displaced femoral neck fractures in the elderly and femoral neck fractures with failed internal fixation); hip replacement for the first time; ability to communicate independently; age ≥ 60 years old. (2) Exclusion criteria involved heart, brain, kidney and hematopoietic dysfunctions; previous history of severe mental illness or delirium; history of Alzheimer’ s disease or epilepsy; previous history of coronary heart disease, hypertension or diabetes mellitus. (3) Elimination criteria contained simultaneous participation in two clinical studies; required termination of trial due to serious adverse reactions during the study. Sixty elderly patients who underwent elective hip replacement were divided into the remimazolam group and the propofol group through the method of random number table. There were no statistically significant differences in the general data between the two groups (*P* > 0.05). This study was approved by the Clinical trial ethics committee of the hospital (2021- scientific research-032) and informed consent was signed by patients or clients.

### Anesthesia methods

Eight h of fasting for food and water was performed on the study subjects before surgery, and venous access was established after entering the room to detect the heart rate, mean arterial pressure (MAP) and blood oxygen saturation, and arterial pressure was measured by puncturing radial artery. Under ultrasound positioning, iliac fascia block was implemented with 40 ml of 0.25% ropivacaine. The block effect was monitored after nerve block and the treatment would be terminated due to poor anesthetic effects (such as failure of block, no significant pain relief, complications of puncture, etc.). 0.4 μg/kg of sufentanil and 0.15 mg/kg of cis-atracurium were used for anesthesia induction, and the remimazolam group was given intravenous injection of 0.2–0.4 mg/kg of remimazolam during induction until loss of consciousness, and the propofol group selected 1.5–2 mg/kg of propofol for intravenous injection and connected to a ventilator after tracheal intubation by selecting constant volume mode of volume-controlled ventilation (VCV), volume tidal (V_T_) of 6–8 ml/kg, inspiratory time/expiratory time (I:E) of 1:2, respiration rate (RR) of 10–16 times/min and end expiratory carbon dioxide partial pressure (P_ET_CO_2_) of 35–45 mmHg. During maintenance of anesthesia, the remimazolam group was maintained by pumping remimazolam at 0.3–0.5 mg/kg/h while the propofol group was maintained by pumping propofol at 4–8 mg/kg/h, and both groups were treated with remifentanil at 0.1–0.25 μg/kg/min for anesthesia maintenance. 0.5 mg of metaraminol or 6 mg of ephedrine were intravenously injected when the intraoperative blood pressure was lower than 20% of the basal blood pressure. Intravenous injection of 0.25 mg of nicardipine was implemented when the blood pressure was higher than 20% of the basal blood pressure. 0.3 mg of atropine or 6 mg of ephedrine were intravenously injected when heart rate was lower than 60 beats/min accompanied by hypotension or heart rate was lower than 50 beats/min lasting more than 1 min. Intravenous injection of 20 mg of esmolol was performed when heart rate was higher than 100 beats/min. The dosages of intravenous drugs were reduced by 20% at 5 min before the end of the surgery, and then the drugs were discontinued. Both remimazolam and propofol group were given 0.3 mg of flumazenil for antagonism after the end of the surgery. When the patients were completely awake, they were sent to the anesthesia recovery room. After 30 min of observation in the anesthesia recovery room, they were sent back to the ward to record the occurrence of postoperative adverse reactions.

### Observation indicators

(1) Anesthetic effects were evaluated by means of comparing the anesthesia-related indicators such as anesthesia time, awakening time and extubation time in the two groups. (2) Analgesic effects of patients were assessed by Visual Analogue Scale (VAS) score [9] during perioperative period (before surgery and at 3 min, 30 min, 60 min and 90 min after surgery). The full score of VAS was 10 points, and the higher the score, the better the analgesic effects. (3) Circulatory indicators such as heart rate and MAP were recorded before anesthesia (T_0_), immediately before laryngeal mask insertion (T_1_), at 5 min after laryngeal mask insertion (T_2_), at 30 min after laryngeal mask insertion (T_3_) and at 5 min after laryngeal mask removal (T_4_). (4) The venous blood of elbow was collected from patients before anesthesia induction and at 24 h after surgery and 72 h after surgery to separate the plasma by conventional centrifugation, and the levels of stress indicators such as plasma epinephrine, norepinephrine and cortisol were detected by radioimmunoassay. (5) Cognitive function of patients was evaluated by Mini-Mental State Examination (MMSE) [10] which consisted of orientation, memory, attention and calculation, recall ability and language ability, with a total score of 27–30 points as normal cognitive function and score < 27 points as cognitive dysfunction. (6) Adverse reactions were recorded.

### Statistical analysis

SPSS22.0 statistical software was used to process the research data. Measurement data conforming to normal distribution were represented by the mean ± standard deviation ($\overline{x}$ ±s), and measurement data between groups were compared by independent sample *t* test. Analysis of variance of repeated measurement data was adopted to compare the measurement data between groups at each time point, and the pairwise comparison between groups was performed by using *LSD-t* test. The enumeration data were described by cases [n (%)] and the between-group comparison was performed by χ^2^ test. *P* < 0.05 was considered that the difference was statistically significant.

### Sample size calculation

It is estimated that the average value of MAP after anesthesia induction in propofol group is 65.2 + 6.8 mmHg, and the average value of MAP in remimazolam group increases by 6.0 mmHg, which have similar variance. Assuming an α value of 0.025, an a β value of 0.1, we calculate 26 participants are required in each group. Considering potential dropout, we increase the sample size of each group to 30 patients.

## Results

### Comparison of general data between the two groups

Sixty patients were enrolled in the study and only 1 case was excluded due to withdrawal, thus 30 cases in the remimazolam group and 29 cases in the propofol group were finally included. There were no statistical differences in the general data such as gender, age, body mass index (BMI) and ASA grading between the two groups of patients (*P* > 0.05), as shown in Table 1.

**Table 1:** Comparison of general data between the two groups [n, ($\overline{x}$ ±s)]

Groups	n	Gender (n)		Age (years old)	BMI (kg/m^2^)	ASA grading (n)		Hip replacement materials (n)		Disease types (n)			
		Male	Female			Grade II	Grade III	Uncemented	Cemented	Hip osteoarthritis	Femoral head necrosis	Traumatic hip arthritis	Other
Remimazolam group	30	11	19	74.31 ± 10.6	24.07 ± 2.17	14	16	21	9	11	8	7	4
Propofol group	29	12	17	75.04 ± 9.98	23.99 ± 2.11	13	16	21	8	10	9	5	5
χ^2^/*t*	–	0.138		0.272	0.144	0.020		0.042		0.534			
*P*	–	0.711		0.787	0.886	0.887		0.838		0.911			

### Anesthetic effects

There were no statistical differences in the anesthesia time, awakening time and extubation time between the remimazolam group and the propofol group (*P* > 0.05), as shown in Table 2.

**Table 2:** Comparison of anesthetic effects ($\overline{x}$ ±s)

Groups	Anesthesia time (min)	Awakening time (min)	Extubation time (min)
Remimazolam group (*n* = 30)	130.16 ± 43.01	1.81 ± 1.10	4.71 ± 1.04
Propofol group (*n* = 29)	131.64 ± 45.63	2.12 ± 1.01	5.02 ± 1.01
*t*	0.128	1.126	1.123
*P*	0.898	0.265	0.266

### Analgesic effects

There were statistically significant differences in the interaction effect and time-point effect of VAS score in the two groups (*P* < 0.05). The VAS scores of the two groups were significantly decreased at each time point after surgery compared with those before surgery (*P* < 0.05), but the difference in the score between the two groups was not statistically significant after surgery (*P* > 0.05), as shown in Table 3.

**Table 3:** Comparison of analgesic effects ($\overline{x}$ ±s)

Groups	VAS score (points)				
	Before surgery	3 min after surgery	30 min after surgery	60 min after surgery	90 min after surgery
Remimazolam group (*n* = 30)	2.91 ± 0.23	1.54 ± 0.16	1.49 ± 0.15	1.45 ± 0.14	1.41 ± 0.16
Propofol group (*n* = 29)	2.89 ± 0.21	1.56 ± 0.15	1.50 ± 0.17	1.46 ± 0.15	1.44 ± 0.15
*F* interaction, *P* interaction	11.061, < 0.001				
*F* time-point, *P* time-point	9.310, < 0.001				
*F* between-group, *P* between-group	1.071, > 0.05				

### Circulation indicators

There were statistically significant differences in the interaction effect, between-group effect and time-point effect of heart rate and MAP in the two groups (*P* < 0.05). The heart rate and MAP at T_1_, T_2_ and T_3_ were significantly reduced in the two groups compared with those at T_0_, but the heart rate and MAP at T_1_, T_2_ and T_3_ in the remimazolam group were significantly higher than those in the propofol group (*P* < 0.05), as shown in Table 4.

**Table 4:** Comparison of circulatory indicators ($\overline{x}$ ±s)

Groups	Heart rate (beats/min)					MAP (mmHg)				
	T_0_	T_1_	T_2_	T_3_	T_4_	T_0_	T_1_	T_2_	T_3_	T_4_
Remimazolam group (*n* = 30)	78.51 ± 11.53	74.1 ± 11.01^a^	71.61 ± 9.92^a^	70.11 ± 8.91^a^	79.52 ± 9.11	100.80 ± 10.21	73.86 ± 9.59^a^	79.41 ± 10.31^a^	85.03 ± 11.02^a^	99.34 ± 12.03
Propofol group (*n* = 29)	76.62 ± 9.31	65.4 ± 8.11	64.63 ± 12.81	62.12 ± 7.34	70.7 ± 11.41	99.41 ± 10.60	64.56 ± 8.34	70.61 ± 9.52	78.41 ± 10.03	97.21 ± 11.64
*F* interaction, *P* interaction	32.134, < 0.001					19.117, < 0.001				
*F* time-point, *P* time-point	21.097, < 0.001					13.085, < 0.001				
*F* between-group, *P* between-group	16.319, < 0.001					8.349, < 0.001				

^a^*P* < 0.05 vs the propofol group; T_0_: before anesthesia induction, T_1_: immediately before laryngeal mask insertion, T_2_: 5 min after laryngeal mask insertion, T_3_: 30 min after laryngeal mask insertion, T_4_: 5 min after laryngeal mask removal

### Stress indicators

The differences in the interaction effect, between-group effect and time-point effect of plasma epinephrine, norepinephrine and cortisol were statistically significant between the two groups (*P* < 0.05). The levels of plasma epinephrine, norepinephrine and cortisol at 24 h after surgery and at 72 h after surgery were significantly enhanced in the two groups compared with those before anesthesia induction, but the levels in the remimazolam group were significantly lower than those in the propofol group (*P* < 0.05), as shown in Table 5.

**Table 5:** Comparison of stress indicators ($\overline{x}$ ±s)

Groups	Epinephrine (μg/mL)			Norepinephrine (μg/mL)			Cortisol (nmol/L)		
	Before anesthesia induction	24 h after surgery	72 h after surgery	Before anesthesia induction	24 h after surgery	72 h after surgery	Before anesthesia induction	24 h after surgery	72 h after surgery
Remimazolam group (*n* = 30)	14.71 ± 1.63	21.31 ± 2.06^a^	19.16 ± 1.97^a^	61.97 ± 6.06	71.64 ± 9.01^a^	68.04 ± 6.99^a^	491.36 ± 48.24	541.16 ± 50.19^a^	521.39 ± 50.22^a^
Propofol group (*n* = 29)	14.32 ± 1.49	31.3 ± 3.05	22.93 ± 2.36	62.07 ± 6.17	91.97 ± 10.99	81.87 ± 8.03	492.47 ± 47.97	630.81 ± 61.14	561.38 ± 52.25
*F* interaction, *P* interaction	67.981, < 0.001			31.617, < 0.001			56.319, < 0.001		
*F* time-point, *P* time-point	29.195, < 0.001			17.311, < 0.001			24.762, < 0.001		
*F* between-group, *P* between-group	33.991, < 0.001			29.696, < 0.001			11.037, < 0.001		

^a^*P* < 0.05 vs the propofol group

### Cognitive function

There were statistically significant differences in the interaction effect, time-point effect and between-group effect of MMSE score in the two groups (*P* < 0.05). The MMSE scores of the two groups were significantly lower at 1 d and 3 d after surgery than those before anesthesia induction, but the scores in the remimazolam group at 1 d and 3 d after surgery were significantly higher than those in the propofol group (*P* < 0.05), as shown in Table 6.

**Table 6:** Comparison of cognitive function ($\overline{x}$ ±s, points)

Groups	Before anesthesia induction	1 d after surgery	3 d after surgery	7 d after surgery
Remimazolam group (*n* = 30)	27.31 ± 2.36	20.64 ± 1.99^a^	22.64 ± 2.31^a^	26.99 ± 2.49
Propofol group (*n* = 29)	26.99 ± 2.41	16.74 ± 1.76	19.37 ± 2.08	25.77 ± 2.51
*F* interaction, *P* interaction	17.079, < 0.001			
*F* time-point, *P* time-point	9.652, < 0.001			
*F* between-group, *P* between-group	11.163, < 0.001			

^a^*P* < 0.05 vs the propofol group

### Adverse reactions

The total incidence rate of adverse reactions in the remimazolam group was significantly lower compared with that in the propofol group (*P* < 0.05), as shown in Table 7.

**Table 7:** Comparison of adverse reactions [n (%)]

Groups	n	Nausea and vomiting	Hypoxemia	Respiratory depression	Emergence agitation	Dizziness	Total incidence
Remimazolam group	30	1 (3.33)	0 (0.00)	0 (0.00)	1 (3.33)	1 (3.33)	3 (10.00)
Propofol group	29	3 (10.34)	1 (3.45)	1 (3.45)	3 (10.34)	2 (6.90)	10 (34.48)
*x*^*2*^	–						5.145
*P*	–						0.023

## Discussion

The main pathological characteristics of elderly patients undergoing hip replacement are degenerative changes of tissues and organs, reduction or atrophy of body cells, declines of body reserve function and compensatory stress ability, and often accompanied by a variety of underlying factors (such as hypertension, diabetes mellitus, coronary heart disease, etc.) and decreased tolerance to anesthesia and surgery [11]. Studies have pointed out that the central nervous system and peripheral receptors in elderly patients are reduced and the drug concentration at the receptor site of each target organ is correspondingly significantly increased, resulting in a significantly enhanced drug effect, and the respiratory and circulatory inhibitory effects of anesthetics in elderly patients are significantly stronger than those of young patients and the drug elimination half-life time is longer in elderly patients [12], therefore how to choose a reasonable and effective anesthesia regimen for elderly patients with hip replacement has become an urgent problem to be solved in clinical application.

Remimazolam is a new type of ultra-short-acting benzodiazepine, which has high affinity with γ-aminobutyric acid receptor and can quickly act on GABA receptor and help the opening of the chloride ion channel, leading to the influx of chloride ion and the hyperpolarization of the nerve cell membrane and then generating obvious anesthetic effects [13], and studies have shown that benzodiazepines can significantly inhibit the inflammatory response of mice and effectively inhibit the concentrations of adrenocorticotropic hormone and cortisol during stress [14]. Propofol, as a short-acting intravenous anesthetic drug, plays a role in the induction and maintenance of general anesthesia, but has obvious inhibitory effects on the respiratory system and circulatory system of patients [15]. Therefore, remimazolam has a better effect in maintaining circulation stability than propofol and it is more advantageous in anesthesia for elderly patients undergoing hip replacement. What’s more, remimazolam has short drug elimination half-life time. Its metabolism is dependent on carboxylesterase 1, with no role for enzymes of the cytochrome P450 superfamily. Although the onset of action and drug interaction of remimazolam is not much more rapid than that of propofol, it has faster drug metabolism [16]. Although propofol has high lipophilicity and can quickly cross the blood-brain barrier to achieve a deep sedative effect in a short period of time, it has been found that propofol can induce a variety of cardiopulmonary complications (such as hypoxia, hypotension, arrhythmia and respiratory depression, etc.) while exerting sedative effects in clinical application, therefore it has certain limitations in the application for elderly patients [17].

Under the dual stimulation of anesthesia and surgery, elderly patients undergoing hip replacement are often in a state of tension and anxiety, with accelerated body circulation, abnormal decreases in heart rate and MAP compared to non-surgical patients, in a state of body’s stress during perioperative period and abnormal expression levels of plasma epinephrine, norepinephrine and cortisol [18]. In 2021, Tang et al. compared the influence of remimazolam and propofol on a variety of inflammatory factors (TNF-α, IL-6, E, COR etc.) during cardiac surgery. In this study, serum IL-6 and TNF-α did not differ preoperatively or 2 h postoperatively between the remimazolam and propofol groups; In 2 h after surgery, the increase of E and COR were significantly lower in the remimazolam group than in the propofol group. Although Tang’s study is different from the operation type, inflammatory index and time point of this study, it still deserves good reference. Their study also claimed that compared with propofol, remimazolam benefited cardiac surgery patients under general anesthesia by reducing hemodynamic fluctuations [19]. Our study showed that there were statistical differences in the heart rate, MAP, plasma epinephrine, norepinephrine and cortisol in the two groups from the aspect of interaction effect, and the heart rate and MAP at T_1_, T_2_ and T_3_ in the two groups were significantly decreased compared with those at T_0_, and the levels of plasma epinephrine, norepinephrine and cortisol in the two groups were significantly increased at 24 h and 72 h after surgery compared with those before anesthesia induction but the levels were significantly lower in the remimazolam group than those in the propofol group, and the heart rate and MAP at T_1_, T_2_ and T_3_ in the remimazolam group were significantly higher than those in the propofol group, indicating that the influence of remimazolam on circulation is smaller than that of propofol during induction and remimazolam can maintain a more stable heart rate and MAP after induction and is more conducive to relieving the stress response of elderly patients during anesthesia. In addition, this study displayed that the VAS scores of the two groups at each time point after surgery were significantly lower than those before anesthesia induction, and there were no significant differences in VAS score, anesthesia time, awakening time and extubation time between the groups, but the awakening time and extubation time of the remimazolam group were slightly shorter than those of the propofol group and the MMSE scores at 1 d and 3 d after surgery were significantly higher than those of the propofol group, preliminarily suggesting that the analgesic effects of remimazolam and propofol are similar in elderly patients with hip replacement but remimazolam may have certain advantages in shortening the awakening time and extubation time and inhibiting the cognitive dysfunction. Both remimazolam and propofol can satisfy the perioperative analgesic effects and effective anesthesia maintenance time of elderly patients, but during the anesthesia process, remimazolam can avoid the excessive and long-lasting sedation that occurs during propofol anesthesia and can have smaller inhibitory effects on the central nervous system of patients.

Currently there are many studies on remimazolam for general anesthesia. For instance, Doi, M., et al. compare the efficacy and safety of remimazolam with propofol for general anesthesia [7]. They find that in terms of recovery time, it is significantly shorter among patients in the propofol group and using flumazenil in remimazolam may offer the opportunity to even surpass the recovery speed of propofol. Overall, a larger percentage of propofol (61.3%) versus remimazolam (41.0%) patients experienced adverse drug reactions, but nausea (7%) and vomiting (6%) in the remimazolam groups were more frequently than that in the propofol group (5.3%) and (4.0%), respectively. Their research is basically consistent with our results, and the difference in the incidence of nausea and vomiting may be caused by the sample size of this experiment, which is much smaller than that of Doi, M., et al.’s research. Remimazolam has the advantages of rapid onset of action, fast metabolism, mild influence on circulation, no disposition to accumulate in long-term application and plays an important role in the induction and maintenance of general anesthesia [20]. Of special note is the availability of a specific antagonist for remimazolam, but not for propofol. The present study revealed that the total incidence rate of adverse reactions in the remimazolam group was significantly lower than that in the propofol group, which was generally consistent with the above-mentioned reports by Doi, M. and others.

In conclusion, remimazolam has similar anesthetic effects as propofol on elderly patients undergoing hip replacement, but the former one has smaller influence on the circulation of body, milder stress response, quicker awakening and higher safety. Generally speaking, general anesthesia with remimazolam may have higher safety in the surgery of elderly patients.

This study has several important limitations. The first is its small sample size and single-center nature. To obtain more reliable conclusions, future studies with larger sample sizes are needed. The second is that there may be a deviation in comparing the anesthetic effects of the two groups after using flumazenil. The last issue isthe experimenter was divided into the remimazolam group and the propofol group through the method of random number table. Anesthesiologists, researchers, and study participants were blinded to allocation. Although the anesthesiologist is blinded to allocation, the appearance of the two general anesthetics is quite different. So they can know the grouping, which may affect the authenticity of the data collected during the operation. But for the data collected after operation, such as the MMSE scores, people who performed the cognitive tests, were not aware of the treatment group of the patient. In addition, MMSE is the most widely used cognitive test, affected by significant ceiling effects and has insufficient sensitivity for detecting mild cognitive impairment and mild dementia, especially in individuals with higher education levels [21]. In the present study, we pay little attention to the patents’ educational backgrounds. These may affect the accuracy of the results.

## Conclusion

We concluded that in elderly patients’ hip replacement, propofol and remimazolam can achieve equivalent anesthetic and analgesic effects; However, remimazolam can significantly relieve respiratory and circulatory suppression, stress response and cognitive dysfunction, more safety.

## Data Availability

The datasets used and/or analysed during the current study available from the corresponding author on reasonable request.

## Abbreviations

VAS: Visual Analogue Scale
MAP: Mean arterial pressure
MMSE: Mini-Mental State Examination
VCV: Volume-controlled ventilation
V_T_: Volume tidal
I:E: Inspiratory time/expiratory time
RR: Respiration rate
P_ET_CO_2_: End expiratory carbon dioxide partial pressure
